# Reply to: Potential contribution of PEP carboxykinase-dependent malate dismutation to the hypoxia response in *C. elegans*

**DOI:** 10.1038/s41467-023-39511-4

**Published:** 2023-07-04

**Authors:** Mehul Vora, Stephanie M. Pyonteck, Tatiana Popovitchenko, Tarmie L. Matlack, Aparna Prashar, Nanci S. Kane, John Favate, Premal Shah, Christopher Rongo

**Affiliations:** 1 The Waksman Institute, Rutgers The State University of New Jersey, Piscataway, NJ 08854 USA; 2 The Department of Genetics, Rutgers The State University of New Jersey, Piscataway, NJ 08854 USA

**Keywords:** Respiration, Gene regulation, Cell signalling

**replying to** R. Comas-Ghierra et al. *Nature Communications* 10.1038/s41467-023-39510-5 (2023)

In a recent paper, we showed that the hypoxia response (HR) pathway promotes gluconeogenesis in *Caenorhabditis elegans* through upregulation of PCK-1, a phosphoenolpyruvate carboxykinase (PEPCK)^1^. In their comment, Comas-Ghierra et al. suggest an additional role for the upregulation PCK-1: driving of the electron transport chain (ETC) through malate dismutation. This is a welcome point, as a complete picture of the different functions of the HR pathway is important for our understanding of oxygen-dependent metabolism.

Oxygen is a terminal electron acceptor for the ETC to continuously receive electrons generated by glycolysis, thereby creating the mitochondrial proton gradient that drives ATP generation. Oxygen deprivation stress (hypoxia) can occur for environmental, physiological, or pathological reasons. One of the roles of the HR pathway is to combat the effects of hypoxia to ensure cellular survival until oxygen is restored. The key effector of the HR pathway is the transcription factor HIFα (HIF-1 in *C. elegans*), which binds to the promoters of target genes and promotes the expression of factors that reprogram metabolism^1, 2^. One well established HIFα functional target from mammalian studies is pyruvate dehydrogenase kinase (PDK1), as upregulation of PDK1 by HIFα reduces flux through the tricarboxylic acid cycle (TCA) and promotes anaerobic ATP generation by glycolysis^3^. However, the complete transcriptional response mediated by HIFα, including how individual gene expression changes are integrated into metabolic and physiological changes, is not well understood.

Our paper provided a complete assessment of HIFα/HIF-1 transcriptional targets and the resulting changes in metabolites when activated under aerobic conditions^1^. Surprisingly, HIF-1 directly upregulates the expression of the PEPCK PCK-1, a key gate keeper for gluconeogenesis. HIF-1 activation also increases metabolites involved in gluconeogenesis and the production of reducing agents like glutathione, suggesting that one of the key facets of the HR pathway is to combat the oxidative stress that occurs in situations like hypoxia. Indeed, we found that the addition of supplemental antioxidants was sufficient to rescue both *hif-1* and *pck-1* mutants from hypoxia-induced damage, demonstrating the importance for this HIF-mediated antioxidant response under hypoxic conditions.

Comas-Ghierra et al. suggest that PCK-1 upregulation might provide an additional mechanism for hypoxia survival: malate dismutation. Nematodes can use malate dismutation to generate ATP under hypoxic conditions through an alternative version of the ETC that uses rhodoquinone (RQ) instead of ubiquinone (UQ) as an electron carrier. PEPCK can contribute to this alternative mechanism, as it can convert PEP to oxaloacetate. Malate dehydrogenase, which is also upregulated by HIF-1, converts oxaloacetate into malate, which can enter the mitochondria to be converted to fumarate in the first step of malate dismutation. Fumarate then takes the place of oxygen as the terminal electron acceptor at complex II of the ETC, allowing for the regeneration of rhodoquinone, which in turn can drive complex I function, proton efflux, and ATP generation. This mechanism has been particularly well studied in parasitic nematodes surviving the anaerobic environment of their host’s intestine, but this pathway also operates in *C. elegans*, which is non-parasitic^4^.

Is HIF-1 promoting malate dismutation by upregulating PCK-1? In gluconeogenesis, the conversion of oxaloacetate to PEP by PEPCK produces CO_2_ and hydrolyzes GTP, two elements of the reaction that make it quite energetically favorable. By contrast, the reverse reaction (conversion of PEP to oxaloacetate) would require elevated levels of dissolved CO_2_ and a high PEP/oxaloacetate ratio. Such conditions would be tissue-specific, dependent on the severity of oxygen deprivation stress, and/or dependent on the level of cellular metabolic activity. It is worth noting that we found that supplementation with PEP, but not oxaloacetate, rescued *hif-1* mutants from hypoxia (Fig. 1a). Moreover, our meta-analysis of human cell line experiments showed that activated HIFα often induces PEPCK expression even though malate dismutation is generally not thought to occur in humans^1^.

**Fig. 1:**
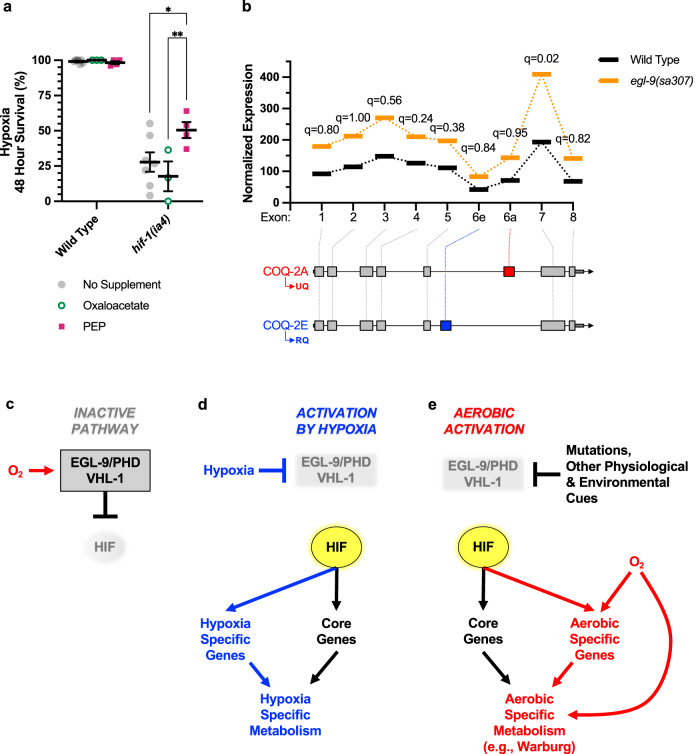
The hypoxia response pathway is a multifunctional context-dependent tool for regulating metabolism. **a** Percent of L4-stage animals surviving hypoxia (0.1% O_2_, 48 h at 25 °C on food, with 24 h recovery at 20 °C) after supplementation with the indicated metabolites. Error bars indicate mean ± SEM. **p = 0.0040, *p = 0.0149 ANOVA/Tukey’s multiple comparison two-sided test between each supplement and the no-supplement control. *N* = 7 biological replicates for untreated wild type and *hif-1* mutant. *N* = 3 biological replicates for oxaloacetate-treated wild type and *hif-1*. *N* = 4 biological replicates for PEP-treated wild type and *hif-1*. Although PEP partially rescued *hif-1* mutants, oxaloacetate, which would hypothetically feed into the malate dismutation pathway downstream of PCK-1, did not. **b** DexSeq analysis of exons from the differentially spliced *coq-2* gene. Isoform COQ-2A is needed to generate UQ under aerobic conditions, whereas hypoxia induces animals to switch to the COQ-2E isoform, which is needed to generate RQ for malate dismutation. No change in isoform ratio was observed in *egl-9* mutants versus wild type under aerobic conditions. Benjamini and Hochberg-corrected likelihood tests for individual exons are indicated by *q*.  **c**–**e** Cartoon models illustrating the multifunctional context-dependent use of the HR pathway. **c** Under aerobic conditions when the pathway is not induced, EGL-9/PHD and VHL-1 use oxygen to inhibit HIF-1. **d** Under hypoxic conditions, HIF-1 is disinhibited, promoting the expression of both core target genes and genes that are activated only under hypoxic conditions. The combination of these upregulated genes modulates metabolism to offset hypoxic damage. **e** Mutations (e.g., in certain cancers) or physiological or environment cues can disinhibit HIF-1 even under aerobic conditions. HIF-1 promotes the expression of both core target genes and genes that are only activated under aerobic conditions. The combination of these upregulated genes modulates metabolism for purposes conducive for aerobic physiology (e.g., the Warburg effect). Source data are provided as a Source Data file.

Another important consideration for answering this question is the presence of oxygen itself. We now have a greater appreciation for how HIFα operates under aerobic conditions (e.g., as part of the Warburg effect), including in cancer cells, dividing stem cells, activated T lymphocytes, endometrial decidualization, patients receiving prolyl hydroxylase inhibitors to treat anemia, and even the flight muscles of locusts^3, 5–14^. We activated HIF-1 by using a null mutant for *egl-9*, as this mutant has constitutively active HIF-1 regardless of oxygen levels. Our choice to study HIF-1 activation under aerobic conditions was intentional, as we expected to find differences in how the pathway regulates gene expression and metabolism depending on whether activation occurs under hypoxic versus aerobic conditions.

Finally, a key component of the malate dismutation mechanism is the regulated alternative splicing of *coq-2*, which encodes a polyprenyl transferase needed to synthesize both UQ and RQ. In an elegant study, Tan et al. showed that hypoxia or potassium cyanide exposure causes nematodes to switch from producing the COQ-2A isoform, which contains exon 6a and generates UQ, to producing the COQ-2E isoform, which contains exon 6e instead of 6a, generating RQ instead of UQ^15^. Although the differential splicing of *coq-2* is an essential facet of the hypoxia response needed for malate dismutation in nematodes, our analysis of exon usage for the *coq-2* transcription unit showed no significant difference when HIF-1 was activated under aerobic conditions (Fig. 1b). Exon 6e, which is a critical exon needed for the COQ-2E isoform that makes RQ, was poorly represented in the transcriptome compared to other exons, and there was no switch in the ratio of exons 6e to 6a. If the malate dismutase pathway is used by the HR pathway under aerobic conditions, then it does not seem to be used as prominently as it is under hypoxic conditions.

We propose that HIFα and the HR pathway are not simply a single survival response to hypoxia, but a multifunctional tool that is employed to reprogram metabolism in diverse ways depending on environmental, developmental, physiological, or pathological context (Fig. 1c–e). A core set of target genes is activated by HIFα regardless of the specific agent activating the pathway, and genes like PEPCK/PCK-1 likely belong to this core. In addition, we speculate that HIFα activates disparate sets of genes that are either tissue dependent or context dependent (e.g., some activated only under aerobic conditions, others activated only under hypoxic conditions). The presence or absence of oxygen likely directs which genes HIFα selects to regulate, operating either through other signaling pathways that crosstalk with the HR pathway, or through oxygen-dependent changes in epigenetic marks. The resulting metabolic changes are also likely to be context dependent, influenced by the metabolites available in specific tissues and/or the availability of oxygen to participate in metabolism. For example, *C. elegans* might use fatty acids shunted through the glyoxylate cycle to fuel gluconeogenesis under aerobic conditions of HIF-1 activation (or post-hypoxia reoxygenation) but not under hypoxic conditions because of the need for oxygen for β-oxidation of fatty acids.

In summary, we agree with Comas-Ghierra et al. that malate dismutation is an additional important adaptation to hypoxia, but we want to highlight that the specific environmental and developmental context of HIFα activation dictates the specific hypoxia response. A next major step in the field will be characterizing this context-dependent activity of HIFα.

## Methods

### Hypoxia survival assays

Nematodes were grown on standard NGM plates containing any indicated supplement prior to collection at the L4 stage for the assay. The supplements included 10 mM oxaloacetate (Sigma O4126) or phosphoenol pyruvic (PEP) acid trisodium salt (VWR IC15187283), which were added to NGM plates, dried at room temperature, then seeded with OP50 bacterial cultures. Garlic extract (100 μL in ethanol) was applied as a ring around the NGM agar bed to prevent nematodes from escaping the dish. L4 stage animals were added to the plates, which were then incubated at 0.1% O_2_ at 25 °C for 48 h with subsequent normoxia recovery at 20 °C for 24 h. No supplement was included in recovery plates. The number of animals alive, dead, or censored (i.e., crawled out of the agar and up the edge of the dish) was counted to generate the percent survival for each trial. Thirty animals per trial were used for the assays, with the average percent of animals surviving for 48 h drawn from at least 3 independent, biological trials.

### Statistical analysis

Simple calculations were done in MS Excel v16.70. Data for survival assays were analyzed using GraphPad Prism 9.5.1. Statistical power analysis was performed for sample size estimations using G*Power v3.1.9.6 based on our own preliminary analysis of mutants at the beginning of the study (or from previous measurements of the mutants as published), where effect size compared to wild type has been large (*d* = 2.3–4.8) depending on the phenotypic assay. Researchers were blind to genotype or experimental treatment. Statistical tests were as indicated in the figure; two-sided tests were used unless otherwise indicated. Data was tested for normality using Kolmogorov-Smirnov and adjusted for multiple comparisons as indicated in the figure legends.

### Transcriptome analysis

Previously published RNA-seq expression data from wild-type and *egl-9(sa307)* mutant L4 stage animals under aerobic conditions was analyzed. Reads were mapped to the *C. elegans* genome and transcriptome (WS273) with STAR 2.5.1a. DexSeq (v 1.28.1) was used to normalize the data and identify genes that have differential exon usage between the two genotypes (FDR < 0.05). Likelihood ratio tests for individual *coq-2* exons were conducted in a pairwise fashion between genotypes with a Benjamini and Hochberg correction. RNA-seq data sets are available at NIH/NCBI GEO through accession number GSE173581.

### Reporting summary

Further information on research design is available in the Nature Portfolio Reporting Summary linked to this article.

## Supplementary information

Reporting Summary

## Data Availability

Files for RNA-seq data sets are available at NIH/NCBI GEO through accession number GSE173581. Files can be directly accessed at the web link GSE173580. The files GSM5271168, GSM5271169, GSM5271176, and GSM527117 contain data for four independent biological replicates for N2 wild-type nematodes. The files GSM5271170, GSM5271171, GSM5271178, and GSM5271179 contain data for four independent biological replicates for *hif-1(ia4)* mutant nematodes. The files GSM5271172, GSM5271173, GSM5271180, and GSM5271181 contain data for four independent biological replicates for *egl-9(sa307)* mutant nematodes. The files GSM5271174, GSM5271175, GSM5271182, and GSM5271183 contain data for four independent biological replicates for *egl-9(sa307) hif-1(ia4)* mutant nematodes. The files GSM5271184, GSM5271185, GSM5271186, and GSM5271187 contain data for four independent biological replicates for OR3350 nematodes. *C. elegans* genome WS273 (WBcel235) was used: https://www.ncbi.nlm.nih.gov/datasets/genome/GCF_000002985.6/. The SuperSeries file of the above subfiles is available at https://www.ncbi.nlm.nih.gov/geo/query/acc.cgi?acc=GSE173581. The source data underlying Fig. 1 is provided online as a single Source Data with this paper. Source data are provided with this paper.

## Source data

Source Data
