# Supplementary material for: Reply to: Potential contribution of PEP carboxykinase-dependent malate dismutation to the hypoxia response in C. elegans
Source: Nat Commun. 2023 Jul 4;14:3937. doi: 10.1038/s41467-023-39511-4 (PMC10319873; doi:10.1038/s41467-023-39511-4)
Supplement: Supplementary file 1 — Reporting Summary [file 41467_2023_39511_MOESM1_ESM.pdf]

nature portfolio

Corresponding author(s): Christopher Rongo
Last updated by author(s): Mar 9, 2023

Reporting Summary

Nature Portfolio wishes to improve the reproducibility of the work that we publish. This form provides structure for consistency and transparency in reporting. For further information on Nature Portfolio policies, see our Editorial Policies and the Editorial Policy Checklist.

Statistics

For all statistical analyses, confirm that the following items are present in the figure legend, table legend, main text, or Methods section.

n/a	Confirmed	
☐	☒	The exact sample size (*n*) for each experimental group/condition, given as a discrete number and unit of measurement
☐	☒	A statement on whether measurements were taken from distinct samples or whether the same sample was measured repeatedly
☐	☒	The statistical test(s) used AND whether they are one- or two-sided<br>*Only common tests should be described solely by name; describe more complex techniques in the Methods section.*
☒	☐	A description of all covariates tested
☐	☒	A description of any assumptions or corrections, such as tests of normality and adjustment for multiple comparisons
☐	☒	A full description of the statistical parameters including central tendency (e.g. means) or other basic estimates (e.g. regression coefficient) AND variation (e.g. standard deviation) or associated estimates of uncertainty (e.g. confidence intervals)
☐	☒	For null hypothesis testing, the test statistic (e.g. *F*, *t*, *r*) with confidence intervals, effect sizes, degrees of freedom and *P* value noted<br>*Give P values as exact values whenever suitable.*
☒	☐	For Bayesian analysis, information on the choice of priors and Markov chain Monte Carlo settings
☒	☐	For hierarchical and complex designs, identification of the appropriate level for tests and full reporting of outcomes
☒	☐	Estimates of effect sizes (e.g. Cohen's *d*, Pearson's *r*), indicating how they were calculated

Our web collection on statistics for biologists contains articles on many of the points above.

Software and code

Policy information about availability of computer code

Data collection	RNA-seq sequencing data was collected using an Illumina HiSeq2000/2500.
Data analysis	Simple calculations were done in MS Excel v16.70. RNA-seq reads were mapped to the genome and counted using STAR 2.5.1a. Normalization and statistical analysis were performed using DexSeq v1.28.1. Data for phenotypic analysis (e.g., survival assays) were analyzed using GraphPad Prism 9.5.1. Specific statistical tests are indicated in the figure legends, including P values. All tests were two-sided unless otherwise indicated. Data normality was tested using Kolmogorov-Smirnov and adjusted for multiple comparisons as indicated in the figure legends. Statistical power analysis was performed for sample size estimations using G*Power v3.1.9.6.

For manuscripts utilizing custom algorithms or software that are central to the research but not yet described in published literature, software must be made available to editors and reviewers. We strongly encourage code deposition in a community repository (e.g. GitHub). See the Nature Portfolio guidelines for submitting code & software for further information.

Data

Policy information about availability of data

All manuscripts must include a data availability statement. This statement should provide the following information, where applicable:

- Accession codes, unique identifiers, or web links for publicly available datasets
- A description of any restrictions on data availability
- For clinical datasets or third party data, please ensure that the statement adheres to our policy

Data supporting the findings of this study are in publicly available repositories.
Files for RNA-seq data sets are available at NIH/NCBI GEO through accession number GSE173581. Files can be directly accessed at the web link https://www.ncbi.nlm.nih.gov/geo/query/acc.cgi?acc=GSE173580. The files GSM5271168, GSM5271169, GSM5271176, and GSM527117 contain data for four independent biological replicates for N2 wild-type nematodes. The files GSM5271170, GSM5271171, GSM5271178, and GSM5271179 contain data for four independent biological replicates for hif-1(ia4) mutant nematodes. The files GSM5271172, GSM5271173, GSM5271180, and GSM5271181 contain data for four independent biological replicates for egl-9(sa307) mutant nematodes. The files GSM5271174, GSM5271175, GSM5271182, and GSM5271183 contain data for four independent biological replicates for egl-9(sa307) hif-1(ia4) mutant nematodes. The files GSM5271184, GSM5271185, GSM5271186, and GSM5271187 contain data for four independent biological replicates for OR3350 nematodes.
C. elegans genome WS273 (WBcel235) was used: https://www.ncbi.nlm.nih.gov/datasets/genome/GCF_000002985.6/
The SuperSeries file of the above subfiles is available at https://www.ncbi.nlm.nih.gov/geo/query/acc.cgi?acc=GSE173581.
Other source data are provided online as a single Source Data with this paper.

Human research participants

Policy information about studies involving human research participants and Sex and Gender in Research.

Reporting on sex and gender	NA
Population characteristics	NA
Recruitment	NA
Ethics oversight	NA

Note that full information on the approval of the study protocol must also be provided in the manuscript.

Field-specific reporting

Please select the one below that is the best fit for your research. If you are not sure, read the appropriate sections before making your selection.

☒ Life sciences ☐ Behavioural & social sciences ☐ Ecological, evolutionary & environmental sciences

For a reference copy of the document with all sections, see nature.com/documents/nr-reporting-summary-flat.pdf

Life sciences study design

All studies must disclose on these points even when the disclosure is negative.

Sample size	Statistical power analysis was performed for sample size estimations using G*Power based on our own preliminary analysis of mutants at the beginning of the study (or from previous measurements of the mutants as published), where effect size compared to wild type has been large (d=2.3-4.8) depending on the phenotypic assay. With an alpha=0.5 and power=0.80, and assuming a modest effect size (d=0.8), we estimated about 30 animals per genotype and condition. Replicate numbers for RNA-seq were chosen based on standard deviation and effect size determined from our previous preliminary data, balanced with the cost of these assays.
Data exclusions	For hypoxia survival assays, animals that desiccated on the side of the dish were censored at the time of their demise.
Replication	Data for phenotypic analysis (e.g., survival assays) were analyzed using at least 3 independent biological replicates, with typically 30 animals assayed per replicate. For data shown, all replicates behaved consistently and reproduced the same finding. RNA-seq was analyzed with 4 biological replicates, all of which behaved consistently and reproduced the same finding.
Randomization	Individual nematodes were sorted into groups to individual Petri dishes based on genotype (assayed by PCR or sequencing). Plates containing nematodes of a given genotype were then picked and exposed to treatment (if applicable) with first examining the animals at the microscopic level. Individual nematodes from each genotype and treatment were then picked randomly from these Petri plates.
Blinding	In all experiments, researchers were blinded to genotype or experimental treatment during the data collection and analysis.

Reporting for specific materials, systems and methods

We require information from authors about some types of materials, experimental systems and methods used in many studies. Here, indicate whether each material, system or method listed is relevant to your study. If you are not sure if a list item applies to your research, read the appropriate section before selecting a response.

Materials & experimental systems

n/a	Involved in the study
☒	☐ Antibodies
☒	☐ Eukaryotic cell lines
☒	☐ Palaeontology and archaeology
☐	☒ Animals and other organisms
☒	☐ Clinical data
☒	☐ Dual use research of concern

Methods

n/a	Involved in the study
☒	☐ ChIP-seq
☒	☐ Flow cytometry
☒	☐ MRI-based neuroimaging

Animals and other research organisms

Policy information about studies involving animals; ARRIVE guidelines recommended for reporting animal research, and Sex and Gender in Research

Laboratory animals	Laboratory strains of C. elegans, all of which are variants of the original N2 strain. Only hermaphrodites were examined, typically at the L4 stage.
Wild animals	No wild animals were used in the study.
Reporting on sex	NA: C. elegans are self-fertilizing hermaphrodites.
Field-collected samples	No field collected samples were used in the study.
Ethics oversight	Not applicable for C. elegans nematodes.

Note that full information on the approval of the study protocol must also be provided in the manuscript.
